# Supplementary material for: A digitalized program to improve antenatal health care in a rural setting in North-Western Burundi: Early evidence-based lessons
Source: PLOS Digit Health. 2023 Apr 17;2(4):e0000133. doi: 10.1371/journal.pdig.0000133 (PMC10109494; doi:10.1371/journal.pdig.0000133)
Supplement: Attachment — Submitted filename: Response to Reviewers - PDIG-D-22-00271.docx [file pdig.0000133.s001.docx]

You are being carbon copied ("cc:'d") on an e-mail "To" "Nadine Misago" [missnad1988@gmail.com](mailto:missnad1988@gmail.com)
CC: "Desire Habonimana" [desire.habonimana@ub.edu.bi](mailto:desire.habonimana@ub.edu.bi), "Roger Ciza" [rogersciza@gmail.com](mailto:rogersciza@gmail.com), "Jean Paul Ndayizeye" [jndayizeye@ughe.org](mailto:jndayizeye@ughe.org), "Joyce Kevin Abalo Kimaro" [joyce.kimaro@giz.de](mailto:joyce.kimaro@giz.de)

PDIG-D-22-00271
A Digitalized Program to Improve Antenatal Health Care in a Rural Setting in North-Western Burundi: Early Evidence-based Lessons
PLOS Digital Health

Dear Dr. Misago,

Thank you for submitting your manuscript to PLOS Digital Health. After careful consideration, we feel that it has merit but does not fully meet PLOS Digital Health's publication criteria as it currently stands. Therefore, we invite you to submit a revised version of the manuscript that addresses the points raised during the review process.

Please submit your revised manuscript within 60 days Jan 06 2023 11:59PM. If you will need more time than this to complete your revisions, please reply to this message or contact the journal office at [digitalhealth@plos.org](mailto:digitalhealth@plos.org). When you're ready to submit your revision, log on to <https://www.editorialmanager.com/pdig/> and select the 'Submissions Needing Revision' folder to locate your manuscript file.

Please include the following items when submitting your revised manuscript:
*       A rebuttal letter that responds to each point raised by the editor and reviewer(s). You should upload this letter as a separate file labeled 'Response to Reviewers'.
*       A marked-up copy of your manuscript that highlights changes made to the original version. You should upload this as a separate file labeled 'Revised Manuscript with Track Changes'.
*       An unmarked version of your revised paper without tracked changes. You should upload this as a separate file labeled 'Manuscript'.

If you would like to make changes to your financial disclosure, please include your updated statement in your cover letter. Guidelines for resubmitting your figure files are available below the reviewer comments at the end of this letter.

We look forward to receiving your revised manuscript.

Kind regards,

Danilo Pani, Ph.D.
Academic Editor
PLOS Digital Health

Journal Requirements:

1. Please send a completed 'Competing Interests' statement, including any COIs declared by your co-authors. If you have no competing interests to declare, please state "The authors have declared that no competing interests exist". Otherwise please declare all competing interests beginning with the statement "I have read the journal's policy and the authors of this manuscript have the following competing interests:"

2. Please provide a/amend your detailed Financial Disclosure statement. This is published with the article. It must therefore be completed in full sentences and contain the exact wording you wish to be published.

a. Please clarify all sources of funding (financial or material support) for your study. List the grants (with grant number) or organizations (with url) that supported your study, including funding received from your institution.
b. State the initials, alongside each funding source, of each author to receive each grant.
c. State what role the funders took in the study. If the funders had no role in your study, please state: “The funders had no role in study design, data collection and analysis, decision to publish, or preparation of the manuscript.”
d. If any authors received a salary from any of your funders, please state which authors and which funders.

If you did not receive any funding for this study, please simply state: “The authors received no specific funding for this work.”

3. Please provide separate figure files in .tif or .eps format only and remove any figures embedded in your manuscript file. Please also ensure that all files are under our size limit of 10MB.

For more information about figure files please see our guidelines:  LINK

<https://journals.plos.org/digitalhealth/s/figures>
<https://journals.plos.org/digitalhealth/s/figures#loc-file-requirements>

4. In the online submission form, you indicated that "Data is available from the first and corresponding author and can be obtained upon valid request". All PLOS journals now require all data underlying the findings described in their manuscript to be freely available to other researchers, either 1. In a public repository, 2. Within the manuscript itself, or 3. Uploaded as supplementary information.
This policy applies to all data except where public deposition would breach compliance with the protocol approved by your research ethics board. If your data cannot be made publicly available for ethical or legal reasons (e.g., public availability would compromise patient privacy), please explain your reasons by return email and your exemption request will be escalated to the editor for approval. Your exemption request will be handled independently and will not hold up the peer review process, but will need to be resolved should your manuscript be accepted for publication. One of the Editorial team will then be in touch if there are any issues.

Additional Editor Comments (if provided):

Although this study is interesting from the public health perspective, there are several points of improvement, as suggested by the Reviewers. Writing must be improved for a publication in a Journal paper. Please consider addressing all of them in the manuscript and in the response letter, providing a rebuttal for the comments you do not want/can accomplish in the revision of the manuscript. Marginal changes to the manuscript cannot be considered a Major Revision, to we will appreciate a substantial revision of your work.


[Note: HTML markup is below. Please do not edit.]

Reviewers' comments:

Reviewer's Responses to Questions

<font color="black"><b>Comments to the Author</b><br><br>
1. Does this manuscript meet PLOS Digital Health’s <a href="<https://journals.plos.org/digitalhealth/s/journal-information#loc-criteria-for-publication>" target="_blank">publication criteria</a>? Is the manuscript technically sound, and do the data support the conclusions? The manuscript must describe methodologically and ethically rigorous research with conclusions that are appropriately drawn based on the data presented.</font>


Reviewer #1: Partly

Reviewer #2: Yes

--------------------

<font color="black">2. Has the statistical analysis been performed appropriately and rigorously?</font>

Reviewer #1: Yes

Reviewer #2: Yes

--------------------

<font color = "black">3. Have the authors made all data underlying the findings in their manuscript fully available (please refer to the Data Availability Statement at the start of the manuscript PDF file)?<br><br> The <a href="<https://journals.plos.org/digitalhealth/s/data-availability>" target="_blank">PLOS Data policy</a> requires authors to make all data underlying the findings described in their manuscript fully available without restriction, with rare exception. The data should be provided as part of the manuscript or its supporting information, or deposited to a public repository. For example, in addition to summary statistics, the data points behind means, medians and variance measures should be available. If there are restrictions on publicly sharing data—e.g. participant privacy or use of data from a third party—those must be specified.</font>

Reviewer #1: No

Reviewer #2: Yes

--------------------

<font color = "black">4. Is the manuscript presented in an intelligible fashion and written in standard English?<br/><br/>PLOS Digital Health does not copyedit accepted manuscripts, so the language in submitted articles must be clear, correct, and unambiguous. Any typographical or grammatical errors should be corrected at revision, so please note any specific errors here.</font>

Reviewer #1: Yes

Reviewer #2: Yes

--------------------

<font color = "black">5. Review Comments to the Author<br/><br/>Please use the space provided to explain your answers to the questions above. You may also include additional comments for the author, including concerns about dual publication, research ethics, or publication ethics. (Please upload your review as an attachment if it exceeds 20,000 characters)</font>

Reviewer #1: I would like to thank the editor for including me in the review process for the manuscript entitled "A Digitalized Program to Improve Antenatal Health Care in a Rural Setting in North-Western Burundi: Early Evidence-based Lessons". The research is important however improving acceptability and allowing for phone ownership may be crucial for increasing effectiveness in the long term.  The manuscript is a straightforward attempt that clearly shows the improvement with antenatal care ANC/attendance.  The analysis should be further developed on the cross-sectional survey to understand enrolled mothers’ perceptions and acceptability of the digital innovation. The manuscript writing can be improved to reach publication level. Minimal transitions and restructuring can help reader to follow the analysis. The references are appropriate however need to be strengthen with more current research on acceptability, link to intervention design, improvement of antenatal care and birth outcomes. Please find below comments and suggestions for the improvement of the manuscript.

Thank you for these insightful comments and inputs. Indeed, the level of mobile phone ownership is key determinant of the success of the innovation. In the manuscript, we have updated the section by adding evidence of the increasing ownership level in Burundi including in rural contexts. This was the rationale behind testing the mobile-based technology with the aim to leverage on the rapidly increasing mobile phone penetration to increase antenatal care attendance and boost maternal and neonatal health outcomes. Plus, we have included updated evidence on acceptability and on the correlation between ANC and maternal and neonatal health outcomes with the view to strengthen the manuscript with much more supporting evidence.

Abstract. Update the sentence with the term “side effects” as it is not fully clear in this context.

We have significantly revised the abstract of the manuscript to make it more comprehensive. The sentence that contains the term “side effects” have also been updated.

Author’s summary.  Add the word “to” improve antenatal care attendance.
We have added the word “to” to the sentence and have also revised the whole Author’s summary section.

Author’s summary. “despite mobile phone ownership” is not clear.

We also have updated the sentence.

Page 3, line 7.  Since you are mentioning correlation between ANC and childbirth, this strong point needs to be reflected in the subsequent sections below. 
Page 4, line 44.  In reference to health outcomes.  As stated, the limitations regarding the need for translation into routine practice and evaluation on the impact of health birth outcomes is critical.  More supporting evidence with correlations of quality antenatal care, ANC services and attendance to routine practice and birth outcomes should be added to the introduction/background section and structured as part of this manuscript.

We have expanded the introduction by adding supporting evidence on the correlation between ANC services and attendance and better maternal and neonatal health outcomes.
Page 6, line 74.  I see that you mention that acceptability determines the uptake of a public health intervention however the results from the theoretical framework of acceptability could go more in depth on the constructs. The level and determinants of acceptability shown in Table 1. could be more robust or expanded on as to understand correlation or impact.  Figure 3 (15) can be detailed especially since results stated overall user acceptability is very low.  It is clear that constructs were measured independently and summed up to yield overall acceptability.

We have considerably expanded these sections.

page 7, line 95.  The significant finding with 200% increase can be represented in a more meaningful graphic.  Figure 4 is clear and straightforward. 

page 9, line 116. Expand or re-word “missing to other revenue generated activities”.  This is not fully clear in the context of side effects.

We have reworded the sentence.

Page 10, line 138.  Regarding translation into routine practice.  As stated above, the limitations regarding the need for translation into routine practice and evaluation on the impact of health birth outcomes is critical. I would include more supporting references. We have expanded sections and added many references.

Page 10, line 140.  I agree that a single-arm study without control is not robust enough to confirm attribution of findings however collecting data and nesting a cross sectional survey to understand perceptions and acceptability is very viable. Good for the bottom-up approach being found successful. Thanks for your appreciation. We too agree with you but think it is ok to let readers know that a controlled study might generate much robust evidence.

Page 10, line 148.   Proof of evidence can be referenced from other studies in the literature.  Comparisons of other health interventions and using this data will help further develop the intervention and predict future use. We have added some comparison evidences to strengthen the section.

Reviewer #2: Attention and monitoring for the pregnancy period in Burundi's territories is an issue of particular concern to the public, research, and governments. Careful periodic monitoring of fetal and female health conditions before childbirth can be a useful tool to prevent risk factors and actively act on all known protective factors to promote better health promotion, maternal and newborn health.

Being able to educate, raise awareness and engage even women located in rural areas and consequently more distant from the main urban centers is certainly an excellent screening tool useful to health centers located in the area and, secondarily, promote greater inclusion to urban realities and consequently better development. This is why I have more interest of further exploration in non-urban or at most semi-urban territories than what was suggested by the participant in the health conference, as the rural context is probably still the one at greatest risk.
The ANC guidelines are for this reason suggested by the WHO for a positive pregnancy experience. I found the application of digital technologies (smartphones) to improve access to health interventions very interesting, especially in light of the fact that we are seeing a rapid expansion in the territory of mobile devices that allow better communication with health professionals and emergency channels.

Thinking about making access to visits more effective by reducing the variable given by pregnant women's forgetfulness of appointments is therefore an excellent health promotion tool.

However, reading the article led me to ask questions about which clarification and possible further study in the final version would be interesting.

The first item of interest, in my opinion, is how the sample taken in the study was constructed. A reader might be interested to know:
- whether people were contacted on a voluntary basis,
- whether they were contacted through health facility channels by word-of-mouth or by posting documents,
- whether they were suggested by nursing staff or other possible modalities.

All pregnant women within the study settings are eligible for intervention. However, an informed consent form was sought before enrolment. All women consented to participate (probably because the intervention is not harmful and intends to improve IT skills through practice. Therefore, the sample size is convenient. We open the cohort for a period of three months and closed thereafter. The study duration was limited to 12 months owing to the donor requirements.

It would also be interesting to know whether prior possession of the mobile device was taken into account as a variable. Indeed, it is likely that prior possession (possibly also to be assessed quantitatively in months or years) may positively influence the acceptability of the ANC pathway. A person who has integrated a mobile device (digital friendly) into her daily routine might behave differently than a person who has had it for only a few months or was equipped with the device only on the occasion of this research.
Again with reference to the sample, it would be useful to understand the reasons why some women might not have accepted the intervention. Mention was made of work-care time balance, but were there other possible side effects reported by women? Did the women who did not accept complain about difficulties in accessibility to the device (e.g., difficulty in reading, manual dexterity, using a very small device, etc.) or was it simply very different from their usual communication tools? We did not assess the reasons why some women did not accept intervention as slightly beyond the study protocol. Plus, we excepted wide variability of the reasons. All we wanted to test was whether a digital-based intervention can improve outcomes in a rural setting in Burundi. Mobile ownership is rapidly expanding, and majority of Burundians have access to it. It would therefore be reasonably fine to capitalise on this technology expansion to scale up proof of evidence.

The second element of interest is the possibility, which has already been rightly considered in the paper by the researchers, of considering a more complex statistical study that could also control for the prior possession/non-possession variable. The integration and randomization of multiple groups, this time also composed of women who were not in possession of personal device but could be given in use in this research, could give the study an additional element of thinking. Finally, again for the statistical part, it would also be of interest to integrate all the data collected by cPanel. Thanks for the insightful contribution. We have acknowledged that randomized and controlled studies would generate stronger evidence. Data captured by the cPanel has been comprehensively reported in this manuscript.

The third element I would explore in more detail, however, is qualitative. Line 68 mentions a live chat for direct contact between nurses and women. It may be an interesting data point to understand:
- what kind of digital tool was used for the live chats,
- whether this device met the elements of usability/accessibility by people,
- whether people requested this kind of service and in which situations,
- how many people requested it,
- what were the topics of the requests made by women,
- whether the people who provided live chat support had been previously trained in the use of this type of device, and
- whether the preference for live chat contact was a consequence of the fact that the women on that day were engaged in work activities away from the medical center and thus were able to get information without moving from their place of work or for other reasons (e.g., shyness, shame about live medical personnel, etc...).
An additional qualitative element that might be of interest to the reader is the active or less active participation of the role of the woman's partner, the father of the future child. Does he not participate totally or has some part participated?

The live chart functionality was useful for questions and answers, if needed. The function allowed mothers and CHWs to post a question which was answered by the health care provider. The live chart was open and visible by every study participant. However, there were no significant (important) charts recorded during the course of the study.

A fourth and final element of interest is to understand what kind of adaptation has been made to the acceptability registration questionnaire and whether this instrument has undergone validation from previous research and how Sekhon's model has been readjusted.

We worked in a collaborative team composed of project implementers (HHNB), donors (GIZ) and partners (MoH) to translate and slightly adapt the framework.

I congratulate the colleagues who carried out this interesting research. I hope the comments may have been helpful.

--------------------

<font color = "black">6. PLOS authors have the option to publish the peer review history of their article (<a href="<https://journals.plos.org/digitalhealth/s/editorial-and-peer-review-process#loc-peer-review-history>" target="_blank">what does this mean?</a>). If published, this will include your full peer review and any attached files.<br><br>

<b>Do you want your identity to be public for this peer review?</b> If you choose “no”, your identity will remain anonymous but your review may still be made public.<br><br>

For information about this choice, including consent withdrawal, please see our <a href="<https://www.plos.org/privacy-policy>" target="_blank">Privacy Policy</a>.</font>


Reviewer #1: Yes: Lisa Catanzaro, MArch, MPH

Reviewer #2: Yes: Andrea Moi

--------------------


[NOTE: If reviewer comments were submitted as an attachment file, they will be attached to this email and accessible via the submission site. Please log into your account, locate the manuscript record, and check for the action link "View Attachments". If this link does not appear, there are no attachment files.]

While revising your submission, please upload your figure files to the Preflight Analysis and Conversion Engine (PACE) digital diagnostic tool, <https://pacev2.apexcovantage.com/>. PACE helps ensure that figures meet PLOS requirements. To use PACE, you must first register as a user. Registration is free. Then, login and navigate to the UPLOAD tab, where you will find detailed instructions on how to use the tool. If you encounter any issues or have any questions when using PACE, please email PLOS at [figures@plos.org](mailto:figures@plos.org). Please note that Supporting Information files do not need this step.
