# Supplementary material for: A digitalized program to improve antenatal health care in a rural setting in North-Western Burundi: Early evidence-based lessons
Source: PLOS Digit Health. 2023 Apr 17;2(4):e0000133. doi: 10.1371/journal.pdig.0000133 (PMC10109494; doi:10.1371/journal.pdig.0000133)
Supplement: Attachment — Submitted filename: Response to Reviewers.docx [file pdig.0000133.s002.docx]

## PLOS Digital Health Decision: Revision required [PDIG-D-22-00271R1] - [EMID:08e827d0bea06dec]

| PLOS Digital Health <em@editorialmanager.com> | Feb 6, 2023, 9:30 AM |  |    |
| --- | --- | --- | --- |
| to me  | | |  |

CC: "Desire Habonimana" [desire.habonimana@ub.edu.bi](mailto:desire.habonimana@ub.edu.bi), "Roger Ciza" [rogersciza@gmail.com](mailto:rogersciza@gmail.com), "Jean Paul Ndayizeye" [jndayizeye@ughe.org](mailto:jndayizeye@ughe.org), "Joyce Kevin Abalo Kimaro" [joyce.kimaro@giz.de](mailto:joyce.kimaro@giz.de)

PDIG-D-22-00271R1
A Digitalized Program to Improve Antenatal Health Care in a Rural Setting in North-Western Burundi: Early Evidence-based Lessons
PLOS Digital Health

Dear Dr. Misago,

Thank you for submitting your manuscript to PLOS Digital Health. After careful consideration, we feel that it has merit but does not fully meet PLOS Digital Health's publication criteria as it currently stands. Therefore, we invite you to submit a revised version of the manuscript that addresses the points raised during the review process.

Please submit your revised manuscript within 30 days Mar 08 2023 11:59PM. If you will need more time than this to complete your revisions, please reply to this message or contact the journal office at [digitalhealth@plos.org](mailto:digitalhealth@plos.org). When you're ready to submit your revision, log on to <https://www.editorialmanager.com/pdig/> and select the 'Submissions Needing Revision' folder to locate your manuscript file.

Please include the following items when submitting your revised manuscript:
*       A rebuttal letter that responds to each point raised by the editor and reviewer(s). You should upload this letter as a separate file labeled 'Response to Reviewers'.
*       A marked-up copy of your manuscript that highlights changes made to the original version. You should upload this as a separate file labeled 'Revised Manuscript with Track Changes'.
*       An unmarked version of your revised paper without tracked changes. You should upload this as a separate file labeled 'Manuscript'.
If you would like to make changes to your financial disclosure, please include your updated statement in your cover letter. Guidelines for resubmitting your figure files are available below the reviewer comments at the end of this letter.

We look forward to receiving your revised manuscript.

Kind regards,

Danilo Pani, Ph.D.
Academic Editor
PLOS Digital Health

Journal Requirements:

1. Please review your reference list to ensure that it is complete and correct. If you have cited papers that have been retracted, please include the rationale for doing so in the manuscript text, or remove these references and replace them with relevant current references. Any changes to the reference list should be mentioned in the rebuttal letter that accompanies your revised manuscript. If you need to cite a retracted article, indicate the article's retracted status in the References list and also include a citation and full reference for the retraction notice. We have reviewed the refence list and updated references which have been retracted or cannot be found. We also have added recent evidence as suggested by one of the reviewers.

Additional Editor Comments (if provided):

According to the Reviewers' comments, the manuscript was improved but there are still some unclear points. I kindly ask the authors to solve, if possible, these issues in order to proceed with the final acceptance.


[Note: HTML markup is below. Please do not edit.]

Reviewers' comments:

Reviewer's Responses to Questions

<font color="black"><b>Comments to the Author</b><br /><br />1. If the authors have adequately addressed your comments raised in a previous round of review and you feel that this manuscript is now acceptable for publication, you may indicate that here to bypass the “Comments to the Author” section, enter your conflict of interest statement in the “Confidential to Editor” section, and submit your "Accept" recommendation.</font>

Reviewer #1: All comments have been addressed

Reviewer #2: All comments have been addressed

--------------------

<font color="black">2. Does this manuscript meet PLOS Digital Health’s <a href="<https://journals.plos.org/digitalhealth/s/journal-information#loc-criteria-for-publication>" target="_blank">publication criteria</a>? Is the manuscript technically sound, and do the data support the conclusions? The manuscript must describe methodologically and ethically rigorous research with conclusions that are appropriately drawn based on the data presented.</font>

Reviewer #1: Yes

Reviewer #2: Yes

--------------------

<font color="black">3. Has the statistical analysis been performed appropriately and rigorously?</font>

Reviewer #1: Yes

Reviewer #2: Yes

--------------------

<font color = "black">4. Have the authors made all data underlying the findings in their manuscript fully available (please refer to the Data Availability Statement at the start of the manuscript PDF file)?<br><br> The <a href="<https://journals.plos.org/digitalhealth/s/data-availability>" target="_blank">PLOS Data policy</a> requires authors to make all data underlying the findings described in their manuscript fully available without restriction, with rare exception. The data should be provided as part of the manuscript or its supporting information, or deposited to a public repository. For example, in addition to summary statistics, the data points behind means, medians and variance measures should be available. If there are restrictions on publicly sharing data—e.g. participant privacy or use of data from a third party—those must be specified.</font>

Reviewer #1: Yes

Reviewer #2: Yes

--------------------

<font color = "black">5. Is the manuscript presented in an intelligible fashion and written in standard English?<br/><br/>PLOS Digital Health does not copyedit accepted manuscripts, so the language in submitted articles must be clear, correct, and unambiguous. Any typographical or grammatical errors should be corrected at revision, so please note any specific errors here.</font>

Reviewer #1: Yes

Reviewer #2: Yes

--------------------

<font color = "black">6. Review Comments to the Author<br/><br/>Please use the space provided to explain your answers to the questions above. You may also include additional comments for the author, including concerns about dual publication, research ethics, or publication ethics. (Please upload your review as an attachment if it exceeds 20,000 characters)</font>

Reviewer #1: The Authors replied well to all the requests for revisions. Results are clear and updated.  In my opinion the paper is ready for publication with minor corrections.

Page 3, line 7: If the surname of the first author of the referenced article is given, "et al." should be shown with a period. We have revised all sections where the referenced article is given as “et al” and added a period (to become “et al."). This has been harmonised everywhere in the manuscript.

Reference section: I am still seeing only 1 article from 2022 and many others are over 5 years old. I am surprised about this in the context of recent technological digital health interventions. This note is very relevant and important to us, thank you so much. We have updated the references with new and recent evidence.

Thank you for your contributions, updates, and I wish you much success with your research.

Reviewer #2: I would like to thank the authors, the reviewer and the editor for the work done between the original submission and this first review, in which many elements are now clearer.
It is worthwhile, however, to try to elaborate further on some elements so that the paper is even more effective and useful for the scientific community and for people who are doing or will do similar research.

In some places in the paper, a total sample size of 132 women is indicated, while in other places the number 131 appears. Specifically, 132 is indicated in the abstract (seventh line) and in line 85, while 131 is indicated in line 169 and in Table 1 line 176. Thank you so much for asking us to clarify this. In fact, we enrolled 132 pregnant women but one of them could not be followed until childbirth. We failed to gain her motivation to continue the program and assumed she willingly dropped out. The analysis was therefore performed on 131 women who completed the follow-up time. We have added this clarification in the revised manuscript.

Source 20 has a link which is no longer active, it was not possible to elaborate on the statement in line 41, a reader might be interested, in addition to what you have rightly pointed out, in understanding how the relationship between drop out at appointments and forgetting scheduled appointments due to lack of reminders and awareness of the importance of the service was detected. It is true, the source has been revamped and can no longer be traced. We have therefore updated the section and included recent evidence to support the claims. Thank you.

Line 73. As also suggested by the editor, the presence of the complete data could be helpful for a better reading of the graphs and statistics. In fact, in addition to the graph in figure 1, which shows the distribution according to the four visits, it would be useful to observe the same type of graph in relation to attendance at individual sessions or even the drop-out percentage of people in relation to having attended one, two or three meetings. Knowing, hypothetically, that the biggest drop out was between the first and second meeting, health promotion measures could be put in place to strengthen women more at that stage. It would also be useful to comment further on the reasons why, according to the authors, the ANC2 line in the graph in figure 1 (line 72) shows very low percentages compared to the 3rd (ANC3) and 4th visit (ANC4).

The above suggestion is very insightful indeed, thank you. However, the baseline observation was solely based on facility-based monthly reports on the percentage of pregnant women who attended ANC meetings, as following:

- ANC1 was estimated based on “expected pregnant women” in the catchment area (which is determined by the statistics office of the MoH)
- ANC2-4 were calculated based on ‘scheduled’ appoints (denominators consisted of the total number of scheduled appointments and the numerators were the total number of pregnant women who show up).

The facility provided attendance rates over a period of 6 months prior to intervention. We therefore do NOT have individual data to enable us detect dropout rates at each stage within the ANC continuum.

line 116. From the above it would appear that we are referring to the WHO recommendation, which, however, states at least eight meetings (line 31). Instead we believe that the authors' intention is to refer to what was the aim of this research, supported by Habonimana et al. i.e. to ensure 4 or more meetings.

Yes, the updated ANC recommendations consist of eight contacts rather than 4 visits. However, the implementation of this updated recommendation had not been effective in many settings including in Burundi (at the time of the study). Therefore, our study outcome was 4 ANC visits as this remained the national recommendation until the date of the study. In fact, until today, the DHIS2 and country reports continue to stick on 4 ANC visits.

line 129 and 226. We are sure that a possible reader would be interested in specifying how Sekhon's model was repurposed and what reflections led to the development of the constructs of satisfaction, attitudes, willingness, ability and side effects from Sekhon's initial writing cited in References, no. 22. Was the questionnaire readapted by changing the original version? If so, it would be useful for the reader to understand which items were changed and how.

We have expanded the section by including a succinct table that highlights the acceptability constructs and the corresponding questions. We hope that this will aid comprehension by the readers.

line 132. The process that led to the binary coding of acceptability is not clear. A possible reader might be interested in understanding whether ranges or a cut-off score were used to determine whether acceptability was to be reported as 1 or 0. We have expanded the section by further explaining how we created the binary outcome which was constructed based on several individual inputs (composite outcome). In fact, some input variables were binary and others scale measurements. We explained how this was done: binary variables took value 1 if the answer is yes, and 0 otherwise. We set a cut-off for scale measurements which took value 1 for positive answers and 0 otherwise. The outcome variable was obtained by combining individual measures which were coded 1 for acceptance or 0, otherwise.

line 145. By mistake the word "Antennal" was reported instead of "Antenatal". Thank you so flagging this error out. Highly appreciated. We have corrected it.

line 229. A reader might be interested in a reflection on how, according to the authors, side-effects could influence acceptability. This is a good idea. However, we did not determine the effect of each construct on the overall acceptability. In fact, overall acceptability is an outcome obtained based on the combination of all individual constructs. Therefore, seeking the effect of one construct on acceptability would imply first computing acceptability without that construct and then modelling the new acceptability on that construct. This would therefore change the framework that was used for analysis. Kindly let us know if this explanation makes sense to you. We are very pleased to discuss further possible options.

Thank you for your cooperation.

--------------------

<font color = "black">7. PLOS authors have the option to publish the peer review history of their article (<a href="<https://journals.plos.org/digitalhealth/s/editorial-and-peer-review-process#loc-peer-review-history>" target="_blank">what does this mean?</a>). If published, this will include your full peer review and any attached files.<br><br>

<b>Do you want your identity to be public for this peer review?</b> If you choose “no”, your identity will remain anonymous but your review may still be made public.<br><br>

For information about this choice, including consent withdrawal, please see our <a href="<https://www.plos.org/privacy-policy>" target="_blank">Privacy Policy</a>.</font>

Reviewer #1: Yes: Lisa Catanzaro

Reviewer #2: Yes: Andrea Moi

--------------------


[NOTE: If reviewer comments were submitted as an attachment file, they will be attached to this email and accessible via the submission site. Please log into your account, locate the manuscript record, and check for the action link "View Attachments". If this link does not appear, there are no attachment files.]

While revising your submission, please upload your figure files to the Preflight Analysis and Conversion Engine (PACE) digital diagnostic tool, <https://pacev2.apexcovantage.com/>. PACE helps ensure that figures meet PLOS requirements. To use PACE, you must first register as a user. Registration is free. Then, login and navigate to the UPLOAD tab, where you will find detailed instructions on how to use the tool. If you encounter any issues or have any questions when using PACE, please email PLOS at [figures@plos.org](mailto:figures@plos.org). Please note that Supporting Information files do not need this step.

__________________________________________________
In compliance with data protection regulations, you may request that we remove your personal registration details at any time.  (Use the following URL: <https://www.editorialmanager.com/pdig/login.asp?a=r>). Please contact the publication office if you have any questions.
